# The ChemicalToolbox: reproducible, user-friendly cheminformatics analysis on the Galaxy platform

**DOI:** 10.1186/s13321-020-00442-7

**Published:** 2020-06-01

**Authors:** Simon A. Bray, Xavier Lucas, Anup Kumar, Björn A. Grüning

**Affiliations:** 1 Department of Computer Science, University of Freiburg, Georges-Köhler-Allee 106, Freiburg, Germany; 2 Roche Pharma Research and Early Development, Roche Innovation Center Basel, F. Hoffmann-La Roche Ltd., Grenzacherstrasse 124, Basel, Switzerland

**Keywords:** Cheminformatics, Protein-ligand docking, QSAR, Galaxy, Molecular dynamics

## Abstract

Here, we introduce the ChemicalToolbox, a publicly available web server for performing cheminformatics analysis. The ChemicalToolbox provides an intuitive, graphical interface for common tools for downloading, filtering, visualizing and simulating small molecules and proteins. The ChemicalToolbox is based on Galaxy, an open-source web-based platform which enables accessible and reproducible data analysis. There is already an active Galaxy cheminformatics community using and developing tools. Based on their work, we provide four example workflows which illustrate the capabilities of the ChemicalToolbox, covering assembly of a compound library, hole filling, protein-ligand docking, and construction of a quantitative structure-activity relationship (QSAR) model. These workflows may be modified and combined flexibly, together with the many other tools available, to fit the needs of a particular project. The ChemicalToolbox is hosted on the European Galaxy server and may be accessed via https://cheminformatics.usegalaxy.eu.

## Introduction

Open-source software packages are now available for a wide range of cheminformatics applications, ranging from downloading [1, 2], manipulating, and processing small molecules [3–5], to protein-ligand docking calculations [6, 7], to quantum chemistry [8]. However, with the growth in the number of applications, the difficulty in combining these tools into easily usable, reproducible analysis workflows increases. Many tools require the user to possess some level of programming skill, or at least ability to use the command line; some also rely on unique file formats. Some tools require compilation of the source code for their use, which not only poses a challenge for computationally inexperienced scientists, but also muddies the waters if another user attempts to reproduce the analysis in another environment [9].

Use of technologies such as Conda [10] and containerization (most notably Docker and Singularity [11–13]) helps to mitigate some of these issues. Conda enables reproducible analyses and simplifies installation, while containerization technologies provide a common working environment across operating systems. However, knowledge of the command line is still required to run software, and the user is responsible for maintaining the thorough records (e.g. through use of a traditional lab book) that are required for full reproducibility of analyses.

Here, we present the ChemicalToolbox, a modular, intuitive platform for cheminformatics analysis, built within the Galaxy system [14, 15]. It combines numerous open-source cheminformatics tools, and integrates them into an intuitive, web-based user interface; requested jobs can then be sent to a high-performace computing (HPC) cluster for execution. Thus, the user has access to a range of useful tools and substantial compute resources, without being exposed directly to the HPC environment, or to the command-line interface used by much cheminformatics software. Tools can be run individually, or combined into workflows, which can then be shared with collaborators. All tools are made publicly available on the European Galaxy server, under the subdomain https://cheminformatics.usegalaxy.eu. As an alternative, the ChemicalToolbox can also be easily installed on personal computers, clusters, and cloud services; once installed, the system can be accessed simultaneously by multiple users, using current standard web browsers.

**Fig. 1:**
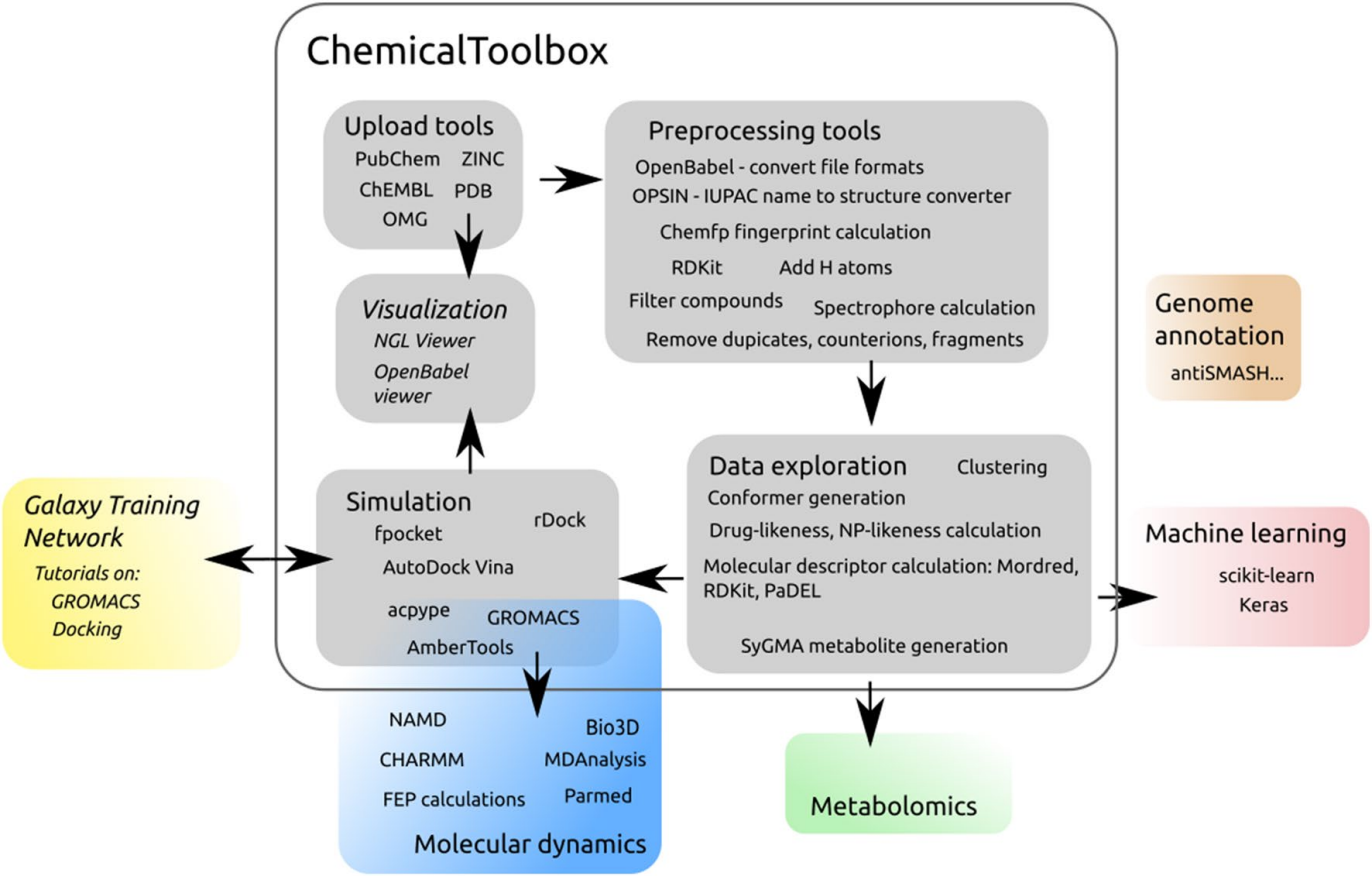
Tools and visualizations available via the ChemicalToolbox. Colored boxes represent other related Galaxy communities, each with their own tools and workflows which can be flexibly combined with those of the ChemicalToolbox

The ChemicalToolbox provides a range of tools for different applications, as depicted in Fig. 1. Chemical structures can be accessed from online databases such as PubChem [2] and ChEMBL [1]. Manipulation of chemical structures can be performed with OpenBabel [4] and RDKit [3], while calculation of molecular descriptors for QSAR studies may be done using Mordred [16] or PaDEL [17], which rely on RDKit and the Chemical Development Kit (CDK) [5] respectively. Protein-ligand docking may be performed using AutoDock Vina [6] and rDock [7]. Furthermore, the previously published BRIDGE platform [18] extends the core functionality of the ChemicalToolbox into molecular dynamics, providing a suite of tools which draws on the GROMACS [19], AmberTools [20], Parmed [21], and MDAnalysis [22] software. Apart from tools, the Galaxy codebase has been extended to provide features particularly useful for cheminformatics. These include support for a range of filetypes commonly used for reporting chemical structures, including PDB, SMILES, InChI, SMILES, SDF/MOL and MOL2, as well as tools for interconverting between these formats, based on OpenBabel. The most common GROMACS filetypes have also been made available. Another feature integrated directly into the Galaxy codebase is the NGLviewer [23], which may be used for visualization of compounds and macromolecules. Furthermore, apart from the features of the ChemicalToolbox itself, the inherent flexibility of the Galaxy system allows combination of the ChemicalToolbox with existing platforms developed by researchers working in other related areas, such as the Galaxy Genome Annotation project, metabolomics (Workflow4Metabolomics [24], Metaboloflow [25]), proteomics (Galaxy-P [26]), and machine learning—enabling the development of new, transdisciplinary workflows.

A number of other workflow management systems are commonly used in cheminformatics; the most prominent are Pipeline Pilot [27] and KNIME [28, 29]. Pipeline Pilot is a workflow management software developed by Accelrys Enterprise Platform and published as a proprietary application. It offers tools bundled into ‘component collections’; two of which, the Chemistry and ADMET collections, provide similar functionality to the ChemicalToolbox. Pipeline Pilot is known for its user-friendly interface and ease of use for new users [30]. However, its proprietary nature makes reproducible research and sharing data very difficult or impossible, and the cost of purchasing a license is prohibitive for many researchers. KNIME, like the ChemicalToolbox, is open-source and free-of-charge, and also leverages well-known open-source software such as the CDK [5, 31] and RDKit in its extensions. KNIME ‘nodes’ are analogous to Galaxy tools, and are assembled into workflows in a similar manner. However, unlike the ChemicalToolbox, the free version of KNIME is not scalable for usage with an HPC or cloud environment; for this, a commercial license for KNIME Server must be purchased. Furthermore, the experience of using KNIME is comparable to programming with a graphical interface; KNIME describes its workflows as a ‘graphic equivalent to a script’. By contrast, the ChemicalToolbox explicitly aims for accessibility to users without programming experience, as the majority of life scientists do not possess these skills.

Offering a cheminformatics toolbox as part of Galaxy has a number of advantages. Firstly, the Galaxy platform is a well-developed, mature project, and while originally developed for genomics research, it is fundamentally agnostic regarding the field of research. The ChemicalToolbox allows chemists to also access the features provided by the Galaxy platform, including a curated body of training material provided by the Galaxy Training Network [32]. Secondly, all ChemicalToolbox tools can be used via the European Galaxy server, which provides free access to generous computational resources for computational analysis, based on the de.NBI cloud [33] and the ELIXIR network [34]. However, the flexibility of the Galaxy system also allows users to download the ChemicalToolbox and run it locally or on their own server. There is already a small but active Galaxy computational chemistry community, constantly maintaining and contributing tools.

## Implementation

While the ChemicalToolbox is primarily available via the European Galaxy instance, it has been designed as a dynamic cheminformatics platform, which can be implemented in diverse working environments and architectures. As it is built on top of the Galaxy framework, the ChemicalToolbox can be configured to run on diverse compute clusters, e.g. Kubernetes [35], TORQUE [36], DRMAA [37], Condor [38], or Pulsar [39]. This scalability allows users to perform compute-intensive cheminformatics calculations, including filtering, converting, and calculating hundreds of physicochemical properties and descriptors for many millions of compounds in a matter of hours.

Any software tool that is parameterizable and can be executed through a terminal command line can be wrapped as a Galaxy tool and included into the ChemicalToolbox, regardless of the programming language used for the implementation of the algorithm. Using the Galaxy ToolShed, each tool can be installed through the user’s web browser by clicking on the required software—analogous to the ‘app stores’ provided by companies such as Apple or Microsoft. Moreover, the associated dependencies are automatically downloaded, compiled, and made accessible within the Galaxy environment. As the Galaxy ToolShed supports tool dependency versioning, the ChemicalToolbox is able to keep track of tool versions, enabling reproducibility and maintaining software provenance over time. Tool execution triggers creation of a Conda environment or download of a container with all software requirements installed, all with the specified versions. When executing outdated workflows in the ChemicalToolbox, the user is notified about newer versions of the tools and is allowed to choose specific versions for execution.

Many kinds of calculations in computational chemistry can be easily parallelized; an example is protein-ligand docking, where each of thousands of compounds in a library needs to be assessed individually. In the ChemicalToolbox, this is achieved by the use of collections. A Galaxy collection allows related files to be grouped together and processed identically. Input files (for example, a docking library in SDF format) are split according to defined parameters (the SDF delimiter), and when the AutoDock Vina or rDock tool is run on the resulting collection, docking is performed for each element of the collection separately and in parallel. Such a parallelization process is carried out automatically in the background, and can be easily parameterized and scaled-up by the server administrator responsible for maintaining the ChemicalToolbox as a suitable platform for high-performance computing.

## Results

Here we present a number of case studies which demonstrate the capabilities of the ChemicalToolbox. For each case study, tools are chained together to form a ‘workflow’, which in the Galaxy system can be used much like an individual tool, thus enabling the flexible creation and combination of new functionalities as desired. Each of the workflows is published online under https://usegalaxy.eu/workflows/list_published and labelled with the ‘cheminformatics’ tag, as are sample Galaxy histories for each of the workflows under https://usegalaxy.eu/histories/list_published. Simplified schematic diagrams of the workflows are provided in Additional file 1, together with individual links to each workflow and history.

### Hole filling and library optimization

The correct choice of chemical libraries is a crucial step in high-throughput virtual screening [40]. By using larger libraries, the chances of identifying hits increase, [41] along with the complexity and resources required for proper storage and testing. Moreover, it has been estimated that the chemical space contains more than $10^{60}$ molecules, a number impossible to handle currently or in the near future [42]. As a consequence, pre-filtered and focused libraries are commonly used in drug discovery, at the risk of exploring a minute portion of the chemical space (from hundreds to millions of compounds) and leaving large regions of the chemical space unexplored. As a result, hole filling and library optimization have assumed a major role in the fields of cheminformatics and drug discovery.

Here we demonstrate a ChemicalToolbox workflow which can be used to optimize a compound library using hole-filling. Downloading all drugs listed on the Therapeutic Target Database [43] (TTD) provides a small library of around 20,000 compounds. For the purpose of this workflow, our aim is to ‘top-up’ this library to 50,000, ensuring that added compounds are located in more sparsely occupied regions of the chemical space. Initially, we download the entirety of the PubChem database, which serves as the source for the new molecules, before calculating molecular fingerprints (using the Chemfp library [44]) for both PubChem and TTD compounds. Taylor-Butina clustering [45] is then performed on the TTD and singletons are identified, i.e. clusters which contain only a single molecule; these are used as seeds for expansion of the compound library. We then perform a similarity search to identify PubChem compounds within a distance threshold of the TTD singletons just found, which yields a total of around 2 million. In order to select compounds evenly, we perform Taylor-Butina clustering once again on our pool of 2 million molecules. A single compound is then selected from each of 30,000 different clusters, and added to the compound library, topping it up to 50,000.

### Ligand library preparation

The preparation of ligand libraries is an important aspect of in silico high-throughput virtual screening, where small molecules are systematically tested in the catalytic or binding site of a protein (for example, via protein-ligand docking) aiming at the selection of candidate compounds with specific structural and physicochemical features. We provide a ChemicalToolbox workflow which offers an efficient solution for the large-scale management of data sets containing millions of molecules.

Initially, the workflow queries several freely available databases (including PubChem, ChEMBL and ZINC [46]) and automatically loads and converts all molecules to canonical SMILES for uniformity using OpenBabel. A specialist tool is used to extract all structures from the PubChem FTP site, while a general download tool can be used to access the other databases. After concatenating the resulting SMILES files and removing counterions and fragments, a final, cleaned dataset of almost 200 million unique compounds in the SMILES format was obtained (databases accessed on 04.10.2019). It is worth mentioning that the ChemicalToolbox has been specifically designed to automatically handle many format files (SDF and SMILES in the present workflow) encoding from a few hundreds or thousands up to many millions of molecules.

### Protein-ligand docking

A common aim in cheminformatics is assessing the interactions of compounds with a protein. Protein-ligand docking involves estimating the interaction energy and the optimal recognition pose of a given ligand in complex with a protein [47, 48]. The ChemicalToolbox contains a number of tools which can be used for protein-ligand docking, including docking software AutoDock Vina and rDock. The fpocket tool can also be used for automatic identification of pockets which are suitable for docking [49].

Firstly, a protein structure and a compound library are created, either uploaded by the user or downloaded directly from online databases such as the PDB or ChEMBL. These can be processed using the Filter tool, which can apply either a commonly-used ruleset, such as Lipinski’s rule-of-five [50], or a set of user-defined properties. In this case, we use two very different systems as illustrative examples: the Hsp90 chaperone protein (structure published under PDB accession code 2brc [51]) and the $\beta _2$-adrenergic receptor (structure published under PDB accession code 3pds [52]). Identification of a binding site allows the definition of a 3D box which is searched (using AutoDock Vina, though rDock is also available) to find a variety of possible binding positions for each of the compounds in the library. Results can be extracted from the output SD files and plotted, or used for further analysis.

### Machine learning for predicting small molecule protein interactions

The Galaxy platform contains tools from multiple disciplines, which offers the opportunity to conduct interdisciplinary analyses. Recently, a suite of statistical and machine learning tools has been made available. This allows the development of quantitative structure-activity relationship (QSAR) models in the ChemicalToolbox.

As an illustrative example, we have published a Galaxy workflow for constructing a random forest classifier for predicting the activity of compounds as agonists of the estrogen receptor alpha signaling (ER$\alpha {}$) pathway. Data are downloaded directly from the relevant PubChem assay, which forms part of the Tox21 program [53]. Initially, tools based on OpenBabel are used to remove counterions or small fragments from the compound library, as well as any duplicated molecules. For the remaining 7459 compounds, over 1800 two- and three-dimensional molecular descriptors are calculated using the Mordred tool [16] and 21 selected as features for building the classification model. A training/test split of 0.7/0.3 was used and a classification model built using the random forest method (in this case, the number of trees used by the classifier is 100) based on the descriptor values calculated for the training data. The random forest algorithm is applied using the implementation published as part of the scikit-learn Python library [54]. Aside from generation of a model that can be applied to new data, the effectiveness of the model can be tested and the results visualized in the form of a ROC curve, precision, recall and f-score plots, and confusion matrix. Here, an AUC value of 0.72 is achieved, which is reasonable considering the simple approach to feature and parameter selection applied here.

### Training material

In addition to publishing the workflows described above, we have also created online tutorials providing an introduction to the features of the ChemicalToolbox, made available via the Galaxy Training Network [32], which already provides a range of introductory and advanced training material for analysis on the Galaxy platform. These tutorials may be found under https://training.galaxyproject.org/training-material/topics/computational-chemistry. For example, the tutorial on protein-ligand docking follows the workflow described above, using a small library of ligands downloaded from ChEMBL and docking them to the Hsp90 protein using AutoDock Vina. In addition, the tutorial guides the user through several other analyses of the compound library, using OpenBabel-based tools to visualize compounds and convert between different formats as required, and performing Taylor-Butina clustering based on calculated chemfp fingerprints.

The Galaxy computational chemistry community has developed a number of other more specialized tutorials, mainly focusing on molecular dynamics simulation and analysis. Other tutorials cover free energy perturbation and the application of machine learning to cheminformatics.

## Conclusions

We have prepared the infrastructure and software for the ChemicalToolbox, a Galaxy-based cheminformatics webserver available via https://cheminformatics.usegalaxy.eu, and made a number of workflows available which demonstrate its capabilities, together with accompanying online introductory tutorials. Such a project can by its nature never be complete or comprehensive; new scientific advances will always result in the development of new software and new analytical approaches. However, the ChemicalToolbox is already sufficiently developed to be used to perform novel and interesting analyses, as well as for pedagogical purposes. We hope that the work published so far will provide a useful resource for chemists and cheminformaticians alike. With this publication, we hope to grow the Galaxy computational chemistry community further and to provide an impetus for further development of the ChemicalToolbox.

## Supplementary information

**Additional file 1.** Additional figures.

## Data Availability

All Galaxy tools are available via the Galaxy ToolShed under https://toolshed.g2.bx.psu.edu and may be executed on the publicly available Galaxy server https://cheminformatics.usegalaxy.eu. Example histories and workflows are available on https://cheminformatics.usegalaxy.eu via the links provided in the article text.
