# Supplementary material for: The ChemicalToolbox: reproducible, user-friendly cheminformatics analysis on the Galaxy platform
Source: J Cheminform. 2020 Jun 1;12:40. doi: 10.1186/s13321-020-00442-7 (PMC7268608; doi:10.1186/s13321-020-00442-7)
Supplement: Supplementary file 1 — Additional file 1. Additional figures. [file 13321_2020_442_MOESM1_ESM.pdf]

Additional material

Supplementary figures S1 to S4 depict simplified schematic diagrams depicting the four workflows discussed in the paper.

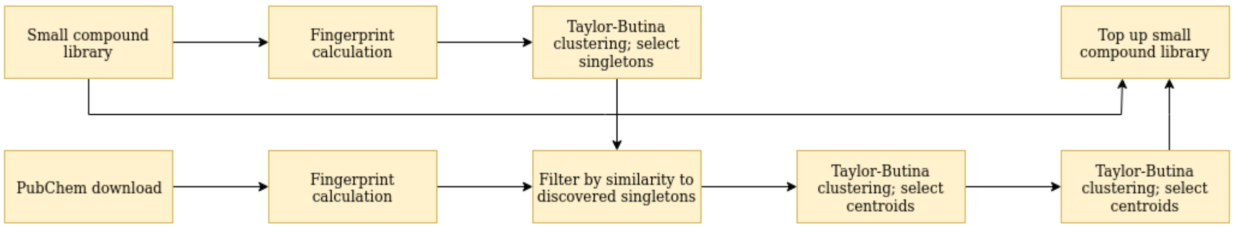

Figure S1. Hole filling.
Workflow: https://cheminformatics.usegalaxy.eu/u/sbray/w/hf080120
Sample history: https://cheminformatics.usegalaxy.eu/u/sbray/h/hole-filling-1

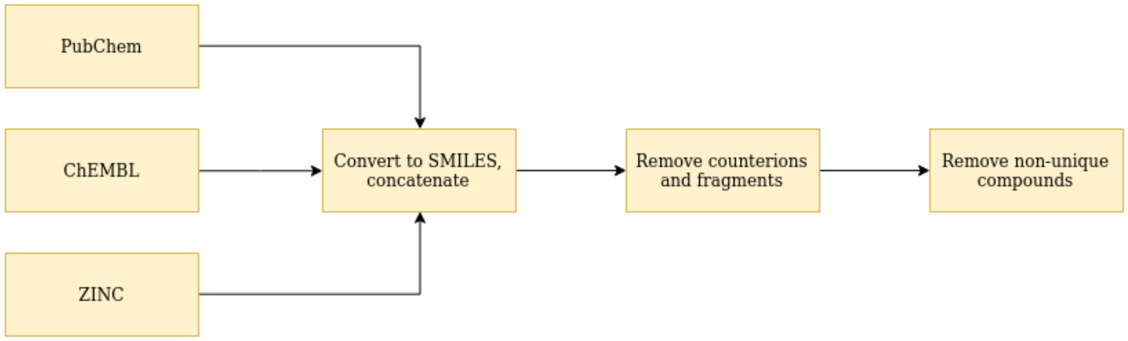

Figure S2. Ligand library preparation.
Workflow: https://usegalaxy.eu/u/sbray/w/library-download-final-2
Sample history: https://usegalaxy.eu/u/sbray/h/compound-library-download

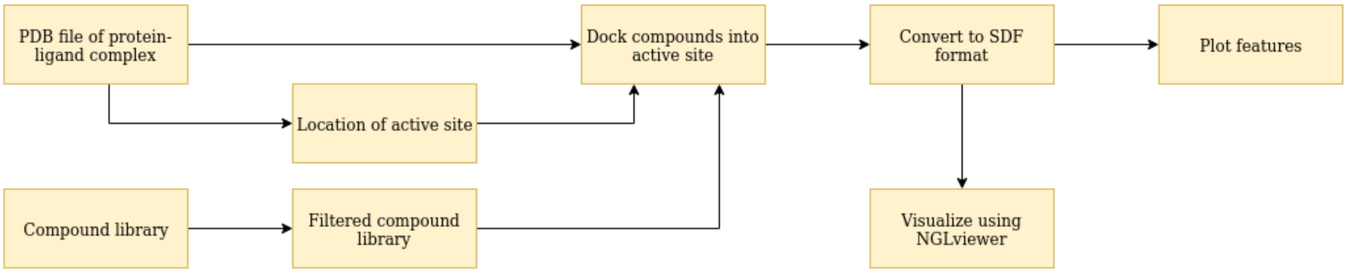

Figure S3. Protein-ligand docking.
Hsp90 workflow: https://cheminformatics.usegalaxy.eu/u/sbray/w/protein-ligand-docking-final
Hsp90 sample history: https://cheminformatics.usegalaxy.eu/u/sbray/h/protein-ligand-docking
B2AR workflow: https://cheminformatics.usegalaxy.eu/u/sbray/w/protein-ligand-docking-b2ar
B2AR sample history: https://cheminformatics.usegalaxy.eu/u/sbray/h/protein-ligand-docking-b2ar

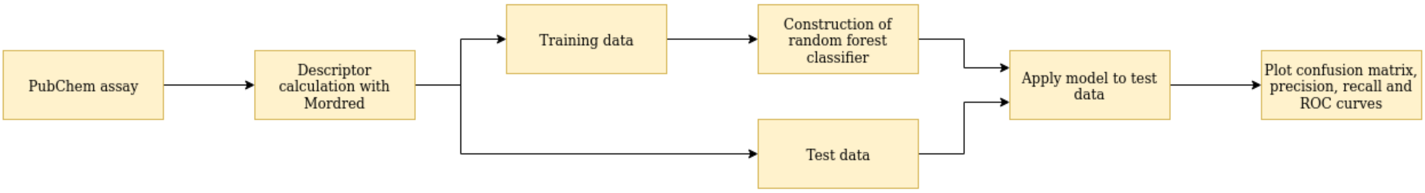

Figure S4. Machine learning for predicting small molecule protein interactions.
Workflow: https://cheminformatics.usegalaxy.eu/u/sbray/w/cheminformatics-ml
Sample history: https://cheminformatics.usegalaxy.eu/u/sbray/h/cheminformatics-ml
